# Surgical Clinic of La Charité

**Published:** 1866-12

**Authors:** 


*Surgical Clinic of La Charité.—Lessons upon the Diagnosis and treatment of Surgical Diseases, delivered in the month of August* 1865—*By Professor Velpeau.*

The above is the title of a small volume of 103 pages, translated by W. C. B. Bifield, M. D., and published by James Campbell, 18 Tremont St., Boston. The List of Contents embraces Generalities, Fractures, Affections of the Joints, Inflammations and Abcesses, Affections of the Lymphatic System, Burns and Contusions, Affections of the Genito-Urinary Organs, Affections of the Anal Region, Affections of the Eye, and Statistics of Operations. We expect benefit in the perusal of anything emanating from this distinguished surgeon.

